# The Influence of Surface Topography and Wettability on *Escherichia coli* Removal from Polymeric Materials in the Presence of a Blood Conditioning Film

**DOI:** 10.3390/ijerph17207368

**Published:** 2020-10-09

**Authors:** I. Devine Akhidime, Anthony J. Slate, Anca Hulme, Kathryn A. Whitehead

**Affiliations:** 1 Microbiology at Interfaces, Manchester Metropolitan University, Chester St, Manchester M1 5GD, UK; D.Akhidime@mmu.ac.uk (I.D.A.); ancajitar@yahoo.com (A.H.); 2 Department of Biology and Biochemistry, University of Bath, Claverton Down, Bath BA2 7AY, UK; ajs319@bath.ac.uk

**Keywords:** Polymer surfaces, *Escherichia coli*, wipe cleaning, sodium hypochlorite, blood conditioning film

## Abstract

The reduction of biofouling and the reduction of cross-contamination in the food industry are important aspects of safety management systems. Polymeric surfaces are used extensively throughout the food production industry and therefore ensuring that effective cleaning regimes are conducted is vital. Throughout this study, the influence of the surface characteristics of three different polymeric surfaces, polytetrafluoroethylene (PTFE), poly(methyl methacrylate) (PMMA) and polyethylene terephthalate (PET), on the removal of *Escherichia coli* using a wipe clean method utilising 3% sodium hypochlorite was determined. The PTFE surfaces were the roughest and demonstrated the least wettable surface (118.8°), followed by the PMMA (75.2°) and PET surfaces (53.9°). Following cleaning with a 3% sodium hypochlorite solution, bacteria were completely removed from the PTFE surfaces, whilst the PMMA and PET surfaces still had high numbers of bacteria recovered (1.2 × 10^7^ CFU/mL and 6.3 × 10^7^ CFU/mL, respectively). When bacterial suspensions were applied to the surfaces in the presence of a blood conditioning film, cleaning with sodium hypochlorite demonstrated that no bacteria were recovered from the PMMA surface. However, on both the PTFE and PET surfaces, bacteria were recovered at lower concentrations (2.0 × 10^2^ CFU/mL and 1.3 × 10^3^ CFU/mL, respectively). ATP bioluminescence results demonstrated significantly different ATP concentrations on the surfaces when soiled (PTFE: 132 relative light units (RLU), PMMA: 80 RLU and PET: 99 RLU). Following cleaning, both in the presence and absence of a blood conditioning film, all the surfaces were considered clean, producing ATP concentrations in the range of 0–2 RLU. The results generated in this study demonstrated that the presence of a blood conditioning film significantly altered the removal of bacteria from the polymeric surfaces following a standard cleaning regime. Conditioning films which represent the environment where the surface is intended to be used should be a vital part of the test regime to ensure an effective disinfection process.

## 1. Introduction

The control of biofouling and the reduction of cross-contamination in the food industry are important aspects of safety management systems. As food products may be contaminated with microbial pathogens at a range of stages during the food production process, cleaning and disinfection are two of the most important hazard control procedures utilised in the food industry [1]. Sodium hypochlorite, a chlorine-releasing compound which acts as a strong oxidiser on the treatment surface, is one of the most widely used sanitisation chemicals in the world and its use is legally defined in some countries, including the USA (Code of Federal Regulations, Title 21) [2]. The main biocidal effect of sodium hypochlorite oxidation is due to the interference with the permeability of microbial cell membranes, coupled with inhibitory action on intracellular enzymes and DNA damage [3,4].

In order for a surface to be hygienic, it needs specific characteristics, such as good cleanability and durability, even in the presence of a conditioning film [5]. Conditioning film formation occurs immediately once the surface is used in its intended environment; this term refers to the adsorption of organic material onto the surface from the surrounding milieu [6]. The adsorption and orientation of the conditioning film onto the surface is dependent on the surface properties [7]. The surface properties are influenced by the interplay of a number of factors, including the surface chemistry, topography and physicochemistry. Previous studies throughout the literature have produced conflicting results on the surface properties that are thought to influence the hygienic status of the surface in the presence of blood. The topographical [8] and surface wettability [9,10,11,12,13,14,15,16] effects of polymers and their influence on surface fouling have been considered, although the overall effects of such surface factors on the hygienic status of the surfaces remain unclear.

Compared to other surface materials such as metals, polymers may be less rigid and have increased malleability [17,18,19]. Over time, polymers can lose their properties, which may cause them to fracture, and this can potentially make them more prone to microbial contamination, due to the formation of surface defects [20]. Polymers may also contain additives to improve their physical properties, which may potentially leach from the surface, providing nutritional support for the attaching microorganisms [21,22,23].

Polymers are used extensively in food packaging, water supply and storage systems and medical applications [24]. In the food industry some of the most commonly utilised polymers include polytetrafluoroethylene (PTFE), poly(methyl methacrylate) (PMMA) and polyethylene terephthalate (PET). PTFE has a white opaque colour and can be manufactured into thin and transparent films. It is flexible, demonstrates a good tensile strength at very low temperatures, has a low friction coefficient [25] and has good chemical stability due to the replacement of hydrogen with fluorine in the C-H bonds [26]. Health concerns have been raised over the use of PTFE due to the use of perfluorooctanoic acid (PFOA; a known carcinogen) in the manufacturing process. However, the production of polymer fumes due to PTFE resin only occurs at temperatures exceeding 260 °C [27]. PTFE provides good electrical insulation and maintains its properties within a large temperature range (−273 °C to 260 °C) and it is regarded as a hygienic surface and its usage extends beyond the food industry [28]. PMMA is often transparent but can have a range of opaque colours. It demonstrates good optical properties and durability and is resistant to inorganic substances, but is soluble in organic substances [29]. PET is transparent and rigid and is an important thermoplastic material due its good malleability [28]. It has a low permeability to water and carbon dioxide and is usually used for electrical/electronic components or bottles used in the food and beverage industry [27,28,29,30,31].

Bacterial cells do not bind directly to the surface substratum, initially they bind reversibly to a conditioning film which can be composed of both organic and inorganic molecules [6]. The chemical structure of a conditioning film depends entirely upon the composition of the surrounding environment, such as macromolecule concentration [32]. Conditioning film composition often includes proteins and carbohydrates, which can influence bacterial attachment by altering the bacterial–surface interface. This can result in enhanced or decreased bacterial attachment and this in turn may affect the amount of resulting biofilm formation [32,33,34]. Therefore, testing surfaces with bacteria in the presence of a relevant fouling material will give a better understanding of bacterial attachment, adhesion and retention mechanisms on polymeric surfaces in situ [33,35].

*Escherichia coli* is a Gram-negative rod and together with coliforms in general, it represents one of the most important indicators of the sanitary quality of water and food surfaces [36,37]. Previous reports suggest that the number of coliforms on a surface should not exceed 25 colony-forming units/cm^2^ (CFU/cm^2^) to be deemed safe for food production [36,38]. Previously, Parent and Velegol (2004), demonstrated that conditioning films could increase *E. coli* colonisation when compared to a clean substratum [39]. The effect of conditioning films (including blood conditioning films) has also been reported to alter surface coatings from non-wettable to wettable [6,31]. This resulted in a reduction in the antimicrobial efficacy of coatings, whilst also reducing the number of retained bacteria on the surface tested [6,32]. Garrido et al. (2014) reported factors such as a decrease in the ionic strength of cells, a reduction in pH and the presence of calcium ions, which influenced *E. coli* retention in the presence of organic conditioning films [40].

Once a surface is installed in a food production/processing area, it will become conditioned with proteins and carbohydrates from the surrounding environment. Although it is known that many studies which try to elucidate the complex interaction between the bacteria and substratum use pristine surfaces, which are not reflective of the intended environment. The addition of a conditioning film can dramatically alter surface topography and physicochemistry, therefore altering bacterial attachment and retention. In light of this, research which aims to understand the underpinning mechanisms of bacterial attachment to food production surfaces should be representative of the environment for which the surfaces are intended; this includes the production of a conditioning film, which is produced by the adsorption of surrounding organic material.

The aim of this study was to determine *E. coli* attachment and retention on three different polymeric surfaces (PTFE, PMMA and PET) in the presence and absence of a blood conditioning film and the impact of this conditioning film was elucidated prior to and post cleaning using a 3% sodium hypochlorite solution.

## 2. Methods

### 2.1. Preparation of Escherichia coli Suspensions

*Escherichia coli* strain NCTC 9001 were cultured on nutrient agar plates (Lab M, UK) at 37 °C for 24 h. Using a sterile metallic loop, colonies of *E. coli* were aseptically transferred into 50 mL of sterile nutrient broth (Lab M, Manchester, UK) and incubated for 24 h at 37 °C. The bacterial suspension was transferred into two sterile universal bottles and centrifuged at 1721× *g* for 10 min. The supernatant was discarded and the cells were recovered into 10 mL sterile water. An optical density (OD_540 nm_) of 1.0 ± 0.1 determined that the bacterial cell concentration in the suspension added to the polymeric surfaces was in the order of ca. 5.0 × 10^8^ CFU/mL. The number of cells that were removed from the surface was established by performing serial dilutions and examining the plates after incubation. Sterile horse blood (TCS Biosciences, Buckingham, UK) was used in the conditioning film assays and was diluted using sterile dH_2_O to 10% (*v*/*v*). Blood and cell samples were mixed in a 1:1 ratio before application to the surfaces, ensuring that the same number of bacteria was applied to each polymer sample.

### 2.2. Surface Wettability

Surface wettability was determined as described in previous work [17,41,42]. Dynamic contact angle analysis was carried out using a DCA 322−1 dynamic angle analyser (Cahn Instruments, Newington, CT, USA). Contact angle measurements of clean and dry substrata were taken using HiPerSolv HPLC-grade water in five-microlitre drops (BDH, Leicestershire, UK). The sample was attached to the balance, immersed, held and then retracted from the solvent.

### 2.3. Optical Interferometry

Optical interferometry was conducted following the methodology of previous work [6]. In order to characterise surface topography, images of each surface were taken using a MicroXAM (phase shift) surface mapping microscope, with an ADE phase shift XYZ 4400 mL system and an AD phase shift controller (Omniscan, Wrexham, UK). The image analysis software used was Mapview AE 2Æ17 (Omniscan, Wrexham, UK). Analysis was carried out using EX mode. The values for *S_a_* (arithmetic mean) for each surface were recorded.

### 2.4. Cleaning Assay

A crockmeter (AATCC, Model CM−1, Stockport, UK) was utilised to clean each of the surfaces during the “wipe clean” study. Samples were attached to microscope slides and ten microlitres of 1.0 ± 0.1 (OD_540 nm_) *E. coli* suspension were deposited on the surface and spread using the tip of a sterile pipette. The samples were placed on the steel holder of the crockmeter. A sterile microfibre cleaning cloth (Vileda Cleaning Microfiber Cloths MicroTuff Lite, Viking Direct, Leicester, UK) with dimensions of 45 × 45 mm was pre-damped with 1.0 mL 3% sodium hypochlorite solution and attached to the test finger. The handle was turned front to back (a total of five times) to replicate a wipe cycle. Afterwards, the steps of swabbing, serial dilution and plating were repeated.

### 2.5. Bacterial Recovery

To determine the number of bacteria recovered from the surface, alginate swabs were moisturised in sterilised water and the polymer surfaces (surface area: 10 mm × 10 mm) were swabbed from left to right and then up and down a total of 10 times. The swab was placed in a Falcon tube containing 10 mL sterilised water, and vortexed for 5 s. The suspension underwent a serial dilution stage and was enumerated by spreading the relevant dilutions onto nutrient agar plates and incubating at 37 °C for 24 h. Total viable counts were calculated from the bacterial colonies isolated.

### 2.6. Percentage Coverage of Bacteria and Organic Material (Blood)

A dual staining method was used to determine the number of cells and conditioning film present on the surface following the cleaning assays. The staining solution consisted of a mixture of DAPI (4’, 6-diamidino-2-phenylindole) (Sigma, Dorset, UK) and rhodamine B ([9-(2-carboxyphenyl)-6-diethylamino-xanthen-3-ylidene]-diethyl-azanium chloride) (Sigma, Dorset, UK) at a 1:1 ratio. The stock concentration for both DAPI and rhodamine B was 0.1 mg/mL. The staining solution (10 µL) was added to the surface and spread evenly across the sample before being dried for 30 min in a class 2 laminar flow cabinet. The samples were examined using an epifluorescence microscope (Nikon Eclipse E600, New York, NY, USA). Images were recorded using a digital camera (Soft Imaging System Ltd., Münster, Germany) and analysed using Cell F Image Analyse package software (Olympus, Tokyo, Japan).

### 2.7. ATP Bioluminescence

The cleaning efficiency and hygienic status of the surfaces, before and after the cleaning regime, were determined by detecting the presence and concentration of adenosine triphosphate (ATP), using a specialised swab system (Ultrasnap^TM^) coupled with a handheld monitoring device (SystemSURE II^TM^ ATP Hygiene Monitoring Device, Hygiena, UK). Under aseptic conditions (ensuring neither the swabs nor the surfaces were handled), the surfaces were swabbed in a consistent manner (as described previously) and the relative light unit (RLU) values were obtained according to the manufacturer’s instructions [35]. An RLU value in the range 0–10 denoted a “clean” surface (little to no ATP present), which would pass food production standards, whilst an RLU > 10 would indicate that the surface needed cleaning and a reading of >30 indicated that the surface was fouled. ATP bioluminescence and the quantification of RLUs has previously been used as an indicator of food surface hygiene [43,44,45,46].

### 2.8. Statistics

Student’s *t*-tests were performed using SPSS (IBM, version 25, New York, NY, USA) to determine significant differences at a confidence level of 95% (*p* < 0.05).

## 3. Results

There was significant variability between the surfaces in terms of their wettability. PTFE was the least wettable surface (118.8°), followed by the PMMA surface (75.2°), whilst the PET surface was the most wettable (53.9°) (Table 1).

Topographical analysis of the substrata identified PTFE as the roughest surface, followed by PMMA and then the PET surface, in all the parameters tested (*S_a_*, maximum feature width and depth, and minimum feature width and depth) (Table 1). Although there was a significant difference between the *S_a_* value and minimum feature depth for the PTFE (0.23 nm) when compared to the other surfaces, there was no difference between the *S_a_* values for the PMMA (0.04 nm) and PET (0.02 nm). There was also no significant difference between the maximum feature width for the PTFE and PMMA. However, there was a statistically significant difference when both PTFE and PMMA were compared to the PET. Each of the polymer surface types demonstrated wide variability when the maximum feature depth and minimum feature width parameters were tested.

**Figure 1:**
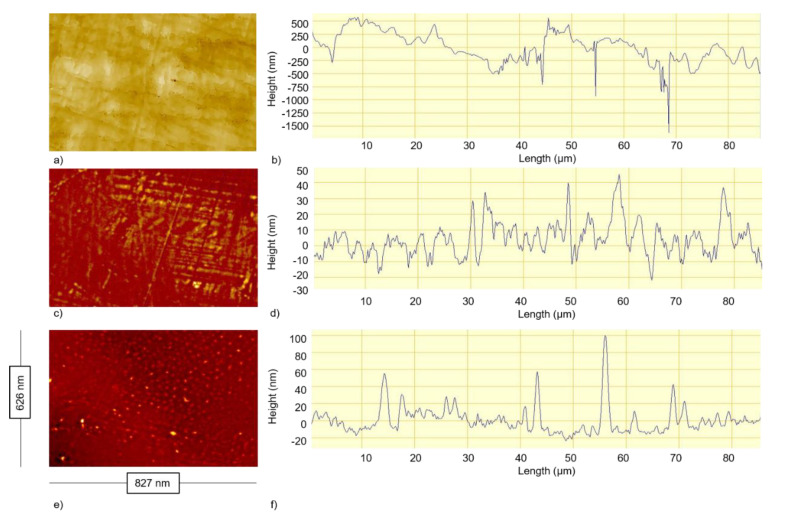
Surface images demonstrating (**a**,**c**,**e**) the overall surface topography and (**b**,**d**,**f**) roughness profiles, demonstrating the differences in the size and shape of the surface features, taken using optical profilometry for (**a**,**b**) PTFE, (**c**,**d**) PMMA and (**e**,**f**) PET.

The changes observed in the roughness values for the surfaces were reflected in the Z profiles using optical profilometry (Figure 1). This demonstrated that the PTFE surface had the greatest difference in the surface features in terms of width and depth (Figure 1a,b), whilst the PMMA surface had the least amount of difference in the surface features with regard to the height irregularities demonstrated (Figure 1c,d). The PET surface revealed the most homogenous surface topography in terms of the height and width of the surface features demonstrated (Figure 1e,f).

**Figure 2:**
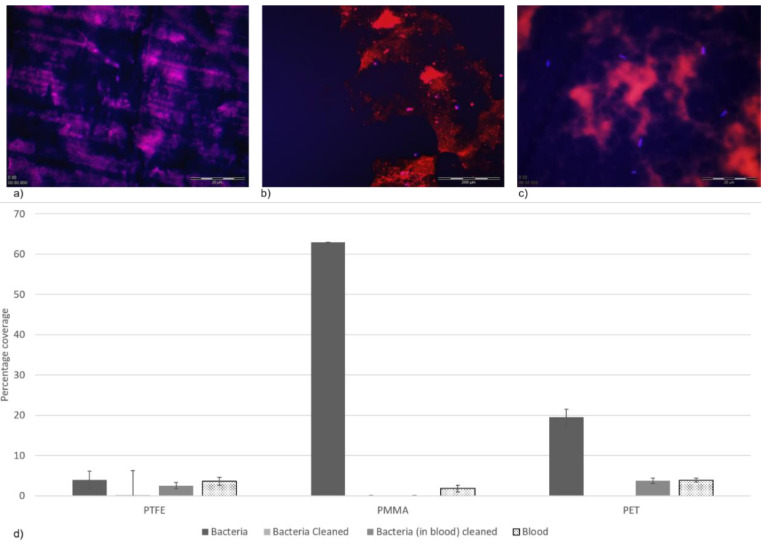
(**a**) Differential staining of the blood (rhodamine B; red) and bacteria (DAPI; blue) across the (**a**) PTFE surface (**b**) PMMA surface and (**c**) PET surface. (**d**) Percentage coverage of the bacteria retained on the surfaces before and after the cleaning regime (3% sodium hypochlorite) both in the presence and absence of a blood conditioning film (*n* = 3).

Prior to cleaning, there was no significant difference in the bacterial load recovered from all three surfaces (*p* > 0.05). Following the cleaning regime of the polymeric surfaces, bacterial enumeration was conducted to observe if the surfaces had any effect on bacterial retention (Table 2). The surface that demonstrated the most cleanable attributes, where no bacterial colonies were recovered, was the PTFE surface. However, although a clear trend was seen, this was not statistically different. This could be due to the rougher surface topography of PTFE (compared to PMMA and PET), which could potentially entrap the bacterial cells, protecting them from the biocidal action (Figure 2d). The bacterial count was 1.2 × 10^7^ CFU/mL for the PMMA surface and was lowest on the PET surface (6.3 × 10^7^ CFU/mL). Interestingly, when the bacteria were applied to the surfaces in the presence of a blood conditioning film, cleaning with 3% sodium hypochlorite resulted in no bacterial colonies being recovered from the PMMA surface. However, on both the PTFE and PET surfaces, bacteria were recovered (2.0 × 10^2^ and 1.3 × 10^3^ CFU/mL, respectively).

The numbers of bacteria recovered from the polymeric surfaces in the presence of a blood conditioning film were evaluated using a differential staining method on the PTFE (Figure 2a), PMMA (Figure 2b) and PET (Figure 2c) surfaces. The results demonstrated that the number of bacterial cells retained on the surfaces, in the presence of a blood conditioning film, was in agreement with the bacterial enumeration results, whereby the PMMA surfaces demonstrated the smallest percentage coverage of bacterial cells (0.07%), followed by PTFE (2.5%), with the most cells retained on the PET surface (3.7%), although the results were not demonstrated to be significantly different. The amount of blood (from the conditioning film) retained on the surface was also not determined to be significantly different. However, the PET (3.7%) surface had a greater percentage coverage of blood than the PTFE (3.6%) and PMMA (1.8%) surfaces.

The ATP bioluminescence assay demonstrated that upon soiling, all the surfaces were considered to need cleaning (>10 RLU). PTFE demonstrated the highest RLU value (132 RLU) (Table 3). Although the same concentration of bacterial cells was applied to all the surfaces, significantly different levels of detection of ATP were determined from all the surfaces analysed (PTFE: 132 RLU, PMMA: 80 RLU and PET: 99 RLU). Following the cleaning regime, the ATP bioluminescence method demonstrated that all the surfaces produced an RLU < 10 and thus were considered clean. The surfaces achieved RLU levels of 0–2 RLU when surfaces were soiled either with the bacteria alone or in the presence of the blood conditioning film.

## 4. Discussion

It is well documented that once a surface is used, it becomes covered with macromolecules (e.g., proteins) from the surrounding environment [7,34]. This study aimed to characterise bacterial adherence to well-known polymeric surfaces (PTFE, PMMA and PET), which are used extensively as food production surfaces, both in the presence and absence of a blood conditioning film, to determine the effect on cleanability, using a wipe clean assay with 3% sodium hypochlorite.

Throughout this study, the PTFE surface was the only surface that was considered non-wettable and it also demonstrated the roughest surface topography. PTFE demonstrated the highest maximum surface feature width and depth measurements and provided evidence that this surface could trap and retain the *E. coli* in the surface features which were a similar size to the dimensions of bacterial cells [6,34]. This could result in a reduction of bacterial cells recovered by conferring protection from shearing forces [47,48]. The PTFE surface achieved the lowest percentage of surface coverage of *E. coli* cells before the cleaning of the surfaces. Following cleaning, the coverage of bacteria on the surfaces significantly dropped, but after cleaning, for the PTFE surface in the presence of blood, the percentage coverage of bacteria increased. This demonstrated that the addition of the blood conditioning film on the PTFE surface enhanced bacterial retention. This may be due to components in the conditioning film binding to the PTFE surface and thus changing the surface properties, resulting in enhanced bacterial retention. With respect to the PTFE surface, this surface was the most non-wettable and roughest surface and retained the most bacteria. Both the rate and extent of bacterial retention are affected by blood conditioning films as the physicochemical parameters of the surface become altered [35,49]. The addition of blood adsorbed onto the surface may have resulted in the surface of the PTFE becoming covered in blood proteins, thus making the surface more wettable, and therefore promoting bacterial attachment.

When a conditioning film was applied to the less wettable PMMA surface, *E. coli* cell retention was not observed (no viable cells were recovered) following 3% sodium hypochlorite treatment. The PET surface was the most wettable surface, and the smoothest surface tested, displaying the lowest surface feature widths and depths. The percentage cell coverage post-application of the cleaning assay revealed a complete removal of the bacterial cells deposited on the PET surface. However, the addition of a blood conditioning film on the PET surface resulted in an increase in *E. coli*. Overall, the addition of a blood conditioning film to the PMMA and PET surfaces resulted in a reduced number of bacteria recovered from the surfaces when compared to the bare substrata. This may simply be the opposite effect of the phenomenon that was demonstrated on the PTFE, whereby the presence of the blood on the surface made them more non-wettable, reducing bacterial attachment. This suggests that there may be optimum surface characteristics that lead to reduced bacterial binding

When a surface comes into contact with blood, plasma proteins adsorb a monolayer on the material within seconds and the protein configuration is dependent on a variety of factors, including the chemical and physicochemical properties of the surface [12,50,51,52]. Albumin has been shown to demonstrate higher adsorption onto PTFE surfaces than fibrinogen [52]. Furthermore, past studies in the authors’ laboratories have demonstrated that albumin can retard bacterial retention on surfaces [31]. Thus, these results suggest that adsorption of a conditioning film to a surface may change the surface properties leading to changes in cleaning efficacy.

Throughout the food industry, the sanitisation standard for microbial contamination reduction on food contact surfaces is generally accepted as 99.999% (a 5-log reduction) achieved in 30 s at room temperature [53]. Therefore, food contact surfaces that may be deemed sanitary may not be completely free of viable microorganisms following cleaning regimes [54]. The total viable count and ATP concentration was determined after a cleaning step, which utilised 3% sodium hypochlorite. ATP bioluminescence measures the concentration of ATP present on a sample as relative light units and is widely used in the food and beverage industries due to its ease of use and fast turnaround times [44,55]. The PTFE surface without a blood conditioning film, which was cleaned with 3% sodium hypochlorite, retained the smallest number of bacterial colonies (no bacterial colonies were observed). However, in the presence of a blood conditioning film, 2.0 × 10^2^ CFU/mL were retained, thus indicating that the conditioning film on the PTFE surface provided protection from the disinfection process. Prior to cleaning, the PTFE surface demonstrated the highest concentration of ATP, however, post cleaning, there was no significant difference in quantified ATP concentration in either in the presence or absence of a conditioning film. This indicated that the ATP quantified prior to the sanitisation process did not correlate with the cell counts observed. The quantification of ATP is an indicator of organic material and is not exclusive to microbial contamination, and whilst studies have shown a correlation between ATP levels and total viable counts, a direct relationship remains unclear [56,57,58].

If microbes survive the sanitisation process, then there is a potential risk of resistance generation to the chemicals used [59]. When this phenomenon is observed, it may be necessary to disinfect or sterilise the contact surface in question to prevent a potentially hazardous build-up of microorganisms. It is important to note that biofilm formation, which can result in bacteria not being exposed to the sanitising chemicals due to extracellular polymeric substance (EPS) production, is not the same problem as biocidal resistance generation. However, when bacterial biofilms are compared to their planktonic counterparts, increased resistance to biocide treatment is conferred [60,61]. Sodium hypochlorite is an effective disinfectant used in the maintenance of food preparation surfaces, and a low concentration (1%) is effective in disinfecting vacutainers within 10 min whilst maintaining good biocompatibility [62]. At higher concentrations (such as 5%), sodium hypochlorite can dissolve bovine pulp tissue (vascularised tissue which is encapsulated in highly mineralised structures) in as little as 20 min [4,63]. In the current study, 3% sodium hypochlorite showed effective disinfection of the PTFE surface without a blood conditioning film and PMMA surfaces with a blood conditioning film. Therefore, the presence of a blood conditioning film resulted in a change in bacterial retention on the polymeric surfaces and an increased concentration of sodium hypochlorite solution could be utilised to degrade the proteinaceous conditioning film on PMMA surfaces, leading to an increase in disinfection efficacy.

The treatment and sanitisation of food contact surfaces in the food industry is directly related to the reduction of microorganisms and, as such, many chemical sanitisers and disinfectants have been studied for their antimicrobial properties on stainless steel surfaces [64,65,66]. This study is in excellent agreement with previous literature, as the sodium hypochlorite treatment had a significant impact on bacterial viability [67,68]. This study highlights the effect of other factors which should be considered when treating food contact surfaces, such as conditioning films. The effect of conditioning films on bacterial retention on a surface was evidenced on the PTFE surface, as the surface properties changed from the most non-wettable surface with the lowest bacterial recovery counts post-cleaning to retaining more bacteria than the most wettable PMMA surface when soiled with a conditioning film.

Throughout this study, the focus has been primarily on planktonic cell phenotypes. However, although in food production environments biofilms are often the main source of microbial contamination [69,70]. Initial microbial binding to a surface is a pre-requisite to biofilm formation and hence it is important to understand such interactions. In future studies, the effect of surface topography, wettability and cleaning regimes should be considered to determine their effects against biofilms. Limitations of this study include bacterial enumeration via CFU/mL, since this method does not account for viable but non-culturable (VBNC) cells [71]. Finally, although ATP quantification is easy to use and rapid, it is an indicator for organic material and not solely microbial contamination [56,57,58].

The efficacy of sodium hypochlorite on bacterial viability is dependent upon the desired outcome. The 3% sodium hypochlorite used in this study demonstrated a 6-log and 5-log reduction on PTFE and PET surfaces, respectively, upon exposure for 2 min. Therefore, if the main aim is to significantly reduce viable bacteria then this is an effective intervention. However, if the objective is to use sodium hypochlorite as a sanitiser or disinfectant on food contact surfaces then the outcome is less clear, as there are certain circumstances where the use of sodium hypochlorite might be less effective, such as PET and PTFE surfaces when in the presence of a blood conditioning film. In order to determine the efficacy of an antimicrobial product, conditioning films similar to those formed in the area where the surface is designed to be used (e.g., food contact surfaces), should be a vital component of the test regime, as surface parameters can be significantly altered.

## 5. Conclusions

The influence of the surface characteristics of three different polymers (polytetrafluoroethylene, PTFE; poly(methyl methacrylate), PMMA; and polyethylene terephthalate, PET) on the removal of *E. coli* from the polymer surfaces using a wipe clean method in the presence of 3% sodium hypochlorite was determined. The presence of a blood conditioning film altered the surface characteristics and therefore had an effect on the retention of *E. coli* on the surfaces post cleaning. Following cleaning with 3% sodium hypochlorite solution, bacteria were completely removed from the PTFE surfaces, whilst the PMMA and PET surfaces still had high numbers of bacteria recovered (1.20 × 10^7^ CFU/mL and 6.3 × 10^7^ CFU/mL, respectively). However, when the bacteria were applied to the surfaces in the presence of a blood conditioning film, cleaning with sodium hypochlorite resulted in no bacteria being recovered from the PMMA surface, whilst on both the PTFE and PET surfaces, bacteria were recovered (at 2.0 × 10^2^ CFU/mL and 1.3 × 10^3^ CFU/mL, respectively). This suggests that the presence of the blood conditioning film had an effect on the efficacy of the sodium hypochlorite, which was dependent on the underlying surface chemistry and wettability of the substrata. To further understand the antimicrobial efficacy of a product, conditioning films similar to those formed in the area where the surface is designed to be used (e.g., food contact surfaces), should be a vital component of the test regime, as the formation of a conditioning film can result in surface parameter alterations which could result in varied cleaning efficacy.

## Figures and Tables

**Table 1:** Contact angles and surface roughness values determined for the polymer surfaces, polytetrafluoroethylene (PTFE), poly(methyl methacrylate) (PMMA) and polyethylene terephthalate (PET) (*n = 3*).

Polymer Material	Contact Angle (°)	*S_a_* (nm)	Max Feature Width (µm)	Max Feature Depth (µm)	Min Feature Width (µm)	Min Feature Depth (µm)
PTFE	118.8°	0.23 (±0.02)	2.94	0.75	0.58	0.05
PMMA	75.2°	0.04 (±0.05)	2.94	0.29	0.09	0.01
PET	53.9°	0.02 (±0.01)	2.35	0.19	0.02	0.01

**Table 2:** Bacterial recovery (CFU/mL) on the polymeric surfaces after cleaning with 3% sodium hypochlorite (*n* = 5).

Polymer Material	Colony-Forming Units (CFU/mL)	
	Surface—No Blood Conditioning Film	Surface—Blood Conditioning Film
**PTFE**	0.0 (±0.0)	2.0 × 10^2^ (±2.4)
**PMMA**	1.2 × 10^7^ (±1.4)	0.0 (±0.0)
**PET**	6.25 × 10^7^ (±3.2)	1.25 × 10^3^ (±1.9)

**Table 3:** Mean values for ATP (relative light unit, RLU) using Ultrasnap^TM^ with a systemSURE II^TM^ device (*n* = 5).

Conditions Tested	ATP Quantification (RLU)		
	PTFE	PMMA	PET
Surfaces prior to cleaning	132 (±29.1)	80 (±12.4)	99 (±31.5)
Surfaces post cleaning	1 (±1.0)	0 (±0.0)	1 (±0.6)
Surfaces with blood conditioning film post cleaning	2 (±3.5)	1 (±1.0)	2 (±1.2)

## References

[1] Van Houdt R., Michiels C.W. (2010). Biofilm formation and the food industry, a focus on the bacterial outer surface. J. Appl. Microbiol..

[2] Food and Drug Administration (2009). Indirect Food Additives: Adjuvants. Production Aids, and Sanitizers. Code of Federal Regulations Title 21.

[3] Bayoumi M.A., Kamal R.M., El Aal S.F.A., Awad E.I. (2012). Assessment of a regulatory sanitization process in Egyptian dairy plants in regard to the adherence of some food-borne pathogens and their biofilms. Int. J. Food Microbiol..

[4] Estrela C., Estrela C.R., Barbin E.L., Spanó J.C.E., Marchesan M.A., Pécora J.D. (2002). Mechanism of action of sodium hypochlorite. Braz. Dent. J..

[5] Verran J., Whitehead K.A. (2006). Assessment of organic materials and microbial components on hygienic surfaces. Food Bioprod. Process..

[6] Slate A., Wickens D., Wilson-Nieuwenhuis J., Dempsey-Hibbert N., West G., Kelly P., Verran J., Banks C.E., Whitehead K.A. (2019). The effects of blood conditioning films on the antimicrobial and retention properties of zirconium-nitride silver surfaces. Colloids Surf. B Biointerfaces.

[7] Saubade F., Akhidime D., Liauw C.M., Benson P., Verran J., Whitehead K.A. (2019). The detection and effect of retained food soils on cell retention on stainless steel surfaces following use in an operational bakery. Food Bioprod. Process..

[8] Minelli C., Kikuta A., Tsud N., Ball M.D., Yamamoto A. (2008). A micro-fluidic study of whole blood behaviour on PMMA topographical nanostructures. J. Nanobiotech..

[9] Yue Y., Hays M.P., Hardwidge P.R., Kim J. (2017). Surface characteristics influencing bacterial adhesion to polymeric substrates. RSC Adv..

[10] Khorasani M.T., Mirzadeh H. (2004). In vitro blood compatibility of modified PDMS surfaces as superhydrophobic and superhydrophilic materials. J. Appl. Pol. Sci..

[11] Desrousseaux C., Sautou V., Descamps S., Traoré O. (2013). Modification of the surfaces of medical devices to prevent microbial adhesion and biofilm formation. J. Hospt. Infect..

[12] Chenga B., Inouea Y., Ishiharaa K. (2019). Surface functionalization of polytetrafluoroethylene substrate with hybrid processes comprising plasma treatment and chemical reactions. Coll. Surf. B: Biointerfaces.

[13] Kodjikian L., Burillon C., Chanloy C., Bostvironnois V., Pellon G., Mari E., Freney J., Roger T. (2002). In vivo study of bacterial adhesion to five types of intraocular lenses. Investig. Ophthalmol. Vis. Sci..

[14] Dou X.Q., Zhang D., Feng C., Jiang L. (2015). Bioinspired hierarchical surface structures with tunable wettability for regulating bacteria adhesion. ACS Nano.

[15] Stallard C.P., McDonnell K.A., Onayemi O.D., O’Gara J.P., Dowling D.P. (2012). Evaluation of protein adsorption on atmospheric plasma deposited coatings exhibiting superhydrophilic to superhydrophobic properties. Biointerphases.

[16] Zhang X., Wang L., Levänen E. (2013). Superhydrophobic surfaces for the reduction of bacterial adhesion. RSC Adv..

[17] Liauw C.M., Slate A.J., Butler J.A., Wilson-Nieuwenhuis J.S.T., Deisenroth T., Preuss A., Verran J., Whitehead K.A. (2020). The Effect of Surface Hydrophobicity on the Attachment of Fungal Conidia to Substrates of Polyvinyl Acetate and Polyvinyl Alcohol. J. Polym. Environ..

[18] Whitehead K.A., Liauw C.M., Wilson-Nieuwenhuis J.S.T., Slate A.J., Deisenroth T., Preuss A., Verran J. (2020). The effect of the surface properties of poly(methyl methacrylate) on the attachment, adhesion and retention of fungal conidia. AIMS Environ. Sci..

[19] Tanaka M., Sato K., Kitakami E., Kobayashi S., Hoshiba T., Fukushima K. (2015). Design of biocompatible and biodegradable polymers based on intermediate water concept. Polym. J..

[20] Jones A.S., Rule J.D., Moore J.S., Sottos N.R., White S.R. (2007). Life extension of self-healing polymers with rapidly growing fatigue cracks. J. R. Soc. Interface.

[21] Whitehead K.A., Verran J. (2009). The effect of substratum properties on the survival of attached microorganisms on inert surfaces. Marine and Industrial Biofouling.

[22] Speranza G., Gottardi G., Pederzolli C., Lunelli L., Canteri R., Pasquardini L., Carli E., Lui A., Maniglio D., Brugnara M. (2004). Role of chemical interactions in bacterial adhesion to polymer surfaces. Biomaterials..

[23] Webb H.K., Crawford R.J., Sawabe T., Ivanova E.P. (2009). Poly(ethylene terephthalate) Polymer surfaces as a substrate for bacterial attachment and biofilm formation. Microbes Environ..

[24] Hahladakis J.N., Velis C.A., Weber R., Iacovidou E., Purnell P. (2018). An overview of chemical additives present in plastics: Migration, release, fate and environmental impact during their use, disposal and recycling. J. Hazard. Mater..

[25] Campo E.A. (2008). Selection of Polymeric Materials: How to Select Design Properties from Different Standards.

[26] Song H., Yu H., Zhu L., Xue L., Wu D., Chen H. (2017). Durable hydrophilic surface modification for PTFE hollow fiber membranes. React. Funct. Polym..

[27] Sajid M., Ilyas M. (2017). PTFE-coated non-stick cookware and toxicity concerns: A perspective. Environ. Sci. Pollut. Res..

[28] Dhanumalayan E., Joshi G.M. (2018). Performance properties and applications of polytetrafluoroethylene (PTFE)—A review. Adv. Compos. Hybrid Mater..

[29] Ali U., Karim K.J.B.A., Buang N.A. (2015). A review of the properties and applications of poly(methyl methacrylate) (PMMA). Polym. Rev..

[30] Ahmad A.F., Razali A.R., Razelan I.S.B.M. (2017). Utilization of polyethylene terephthalate (PET) in asphalt pavement: A review. IOP Conf. Series Mater. Sci. Eng..

[31] Hall C. (2017). Polymer Materials: An Introduction for Technologists and Scientists.

[32] Lorite G.S., Rodrigues C.M., De Souza A.A., Kranz C., Mizaikoff B., Cotta M.A. (2011). The role of conditioning film formation and surface chemical changes on Xylella fastidiosa adhesion and biofilm evolution. J. Colloid Interface Sci..

[33] Dolan R.M. (2002). Biofilms: Microbial life on surfaces. Emerg. Infect. Dis..

[34] Whitehead K.A., Colligon J., Verran J. (2005). Retention of microbial cells in substratum surface features of micrometer and sub-micrometer dimensions. Colloids Surf. B Biointerfaces.

[35] Moreira J., Gomes L.C., Whitehead K.A., Lynch S., Tetlow L., Mergulhão F. (2017). Effect of surface conditioning with cellular extracts on Escherichia coli adhesion and initial biofilm formation. Food Bioprod. Process..

[36] Van Houdt R., Michiels C.W. (2005). Role of bacterial cell surface structures in Escherichia coli biofilm formation. Res. Microbiol..

[37] Rock C.M., Brassill N., Dery J.L., Carr D., McLain J.E., Bright K.R., Gerba C.P. (2019). Review of water quality criteria for water reuse and risk-based implications for irrigated produce under the FDA Food Safety Modernization Act, produce safety rule. Environ. Res..

[38] Moore G., Griffith C. (2002). A comparison of surface sampling methods for detecting coliforms on food contact surfaces. Food Microbiol..

[39] Parent M.-E., Velegol D. (2004). Escherichia coli adhesion to silica in the presence of humic acid. Colloids Surf. B Biointerfaces.

[40] Garrido K.D., Palacios R.J.S., Lee C., Kang S. (2014). Impact of conditioning film on the initial adhesion of E. coli on polysulfone ultrafiltration membrane. J. Ind. Eng. Chem..

[41] Van Oss C.J. (1995). The hydrophilicity and hydrophobicity of clay minerals. Clays Clay Miner..

[42] Van Oss C.J., Chaudhury M.K., Good R.J. (1988). Interfacial Lifshitz-van der Waals and polar interactions in macroscopic systems. Chem. Rev..

[43] Osimani A., Garofalo C., Clementi F., Tavoletti S., Aquilanti L. (2014). Bioluminescence ATP Monitoring for the Routine Assessment of Food Contact Surface Cleanliness in a University Canteen. Int. J. Environ. Res. Public Health.

[44] Omidbakhsh N., Ahmadpour F., Kenny N. (2014). How reliable are ATP bioluminescence meters in assessing decontamination of environmental surfaces in healthcare settings?. PLoS ONE.

[45] Chen F.-C., Godwin S.L. (2006). Comparison of a rapid ATP bioluminescence assay and standard plate count methods for assessing microbial contamination of consumers’ refrigerators. J. Food Prot..

[46] Hygiena Directions for Use of UltrasnapTM ATP Swab with SystemSURE IITM ATP Hygiene Monitoring Device. https://www.hygiena.com/other-products/ultrasnap-other.html.

[47] Al Groosh D.H., Bozec L., Pratten J., Hunt N.P. (2015). The influence of surface roughness and surface dynamics on the attachment of Methicillin-Resistant Staphylococcus aureus onto orthodontic retainer materials. Dent. Mater. J..

[48] Katsikogianni M., Missirlis Y.F. (2004). Concise review of mechanisms of bacterial adhesion to biomaterials and of techniques used in estimating bacteria-material interactions. Eur. Cells Mater..

[49] Kokare C.R., Chakraborty S., Khopade A.N., Mahadik K.R. (2009). Biofilm: Importance and applications. Indian J. Biotechnol..

[50] Engberg A.E., Rosengren-Holmberg J., Chen H., Nilsson B., Lambris J.D., Nicholls I.A., Ekdahl K.N. (2011). Blood protein-polymer adsorption: Implications for understanding complement-mediated hemoincompatibility. J. Biomed. Mater. Res. Part A.

[51] Horbett T.A., Ratner B.D., Schakenraad J.M., Schoen F.J. (2004). Biomaterials Science: An Introduction to Materials in Medicine.

[52] Andersson J., Ekdahl K.N., Larsson R., Nilsson U.R., Nilsson B. (2002). C3 adsorbed to a polymer surface can form an initiating alternative pathway convertase. J. Immunol..

[53] Horwitz W., Latimer G.W. (2005). Official Methods of Analysis.

[54] Tuladhar E., Hazeleger W.C., Koopmans M., Zwietering M.H., Beumer R.R., Duizer E. (2012). Residual viral and bacterial contamination of surfaces after cleaning and disinfection. Appl. Environ. Microbiol..

[55] Bellamy E. (2012). An audit of cleaning effectiveness using adenosine triphosphate (ATP) bioluminescence assay following outbreaks of infection. J. Infect. Prev..

[56] Amodio E., Dino C. (2014). Use of ATP bioluminescence for assessing the cleanliness of hospital surfaces: A review of the published literature (1990–2012). J. Infect. Public Health.

[57] Messina G., Ceriale E., Nante N., Manzi P. (2014). Effectiveness of ATP bioluminescence to assess hospital cleaning: A reviewEmma Ceriale. Eur. J. Public Health.

[58] Sanna T., Dallolio L., Raggi A., Mazzetti M., Lorusso G., Zanni A., Farruggia P., Leoni E. (2018). ATP bioluminescence assay for evaluating cleaning practices in operating theatres: Applicability and limitations. BMC Infect. Dis..

[59] Bolton D., Meally A., Blair I., A McDowell D., Cowan C. (2008). Food safety knowledge of head chefs and catering managers in Ireland. Food Control.

[60] Bridier A., Briandet R., Thomas V., Dubois-Brissonnet F. (2011). Resistance of bacterial biofilms to disinfectants: A review. Biofouling.

[61] Slate A., Shalamanova L., Akhidime D., Whitehead K.A. (2019). Rhenium and yttrium ions as antimicrobial agents against multidrug resistant Klebsiella pneumoniae and Acinetobacter baumannii biofilms. Lett. Appl. Microbiol..

[62] Kukanur S., Nagaraj C., Latha G. (2018). Study of the effectiveness of 1 % sodium hypochlorite on blood samples discarded in a clinical laboratory. Int. J. Curr. Microbiol. Appl. Sci..

[63] Grossman L.I., Meiman B.W. (1941). Solution of pulp tissue by chemical agents. J. Am. Dent. Assoc..

[64] Verran J., Boyd R.D., Hall K., West R.H. (2001). Microbiological and Chemical Analyses of Stainless Steel and Ceramics Subjected to Repeated Soiling and Cleaning Treatments. J. Food Prot..

[65] Verran J., Whitehead K.A. (2005). Factors affecting microbial adhesion to stainless steel and other materials used in medical devices. Int. J. Artif. Organs.

[66] Cabeça T.K., Pizzolitto A.C., Pizzolitto E.L. (2012). Activity of disinfectants against foodborne pathogens in suspension and adhered to stainless steel surfaces. Braz. J. Microbiol..

[67] Sarjit A., Dykes G.A. (2017). Antimicrobial activity of trisodium phosphate and sodium hypochlorite against Salmonella biofilms on abiotic surfaces with and without soiling with chicken juice. Food Control.

[68] Phillips C.A. (2016). Bacterial biofilms in food processing environments: A review of recent developments in chemical and biological control. Int. J. Food Sci. Technol..

[69] Chmielewski R., Frank J. (2003). Biofilm formation and control in food processing facilities. Compr. Rev. Food Sci. Food Saf..

[70] Møretrø T., Langsrud S. (2004). Listeria monocytogenes: Biofilm formation and persistence in food-processing environments. Biofilms.

[71] Cundell T. (2015). The limitations of the colony-forming unit in microbiology. Eur. Pharm. Rev..

